# Marked Decline in Revision Rate Over Time After Hip Arthroscopy for Femoroacetabular Impingement Syndrome

**DOI:** 10.1177/23259671251326112

**Published:** 2025-05-20

**Authors:** David Erlandsson, Philip Quist, Ida Lindman, Axel Öhlin, Louise Karlsson, Nicklas Olsson, Mikael Sansone, Neel Desai

**Affiliations:** † Sahlgrenska Sports and Trauma Research Center, Institute of Clinical Sciences, Sahlgrenska Academy, University of Gothenburg, Gothenburg, Sweden; ‡ Department of Orthopedics, Sahlgrenska University Hospital, Gothenburg, Sweden; Investigation performed at Sahlgrenska Sports and Trauma Research Center, Institute of Clinical Sciences, Sahlgrenska Academy, University of Gothenburg, Gothenburg, Sweden

**Keywords:** femoroacetabular impingement, femoroacetabular impingement syndrome, hip arthroscopy, patient-reported outcome measure, revision rate

## Abstract

**Background:**

There is a body of scientific evidence showing good short-term outcomes after arthroscopic treatment for femoroacetabular impingement syndrome (FAIS); however, less is known about the change in short-term outcomes over time.

**Purpose:**

To (1) evaluate short-term outcomes after arthroscopic treatment for FAIS between 2 groups with index surgery at different periods and (2) assess whether there has been a change in clinical markers over time.

**Study Design:**

Cohort study; level of evidence, 3.

**Methods:**

Data were retrospectively reviewed from a local hip arthroscopy registry, with primary hip arthroscopic treatment for FAIS and age ≥18 years as the inclusion criteria. The exclusion criteria consisted of surgical intervention to either hip before the primary arthroscopic intervention of the studied hip. Patients who underwent treatment between 2012 and 2013 were compared with patients who underwent treatment between 2017 and 2018. The revision rate within 2 years postoperatively was used as the primary outcome. Changes in patient-reported outcome measures (PROMs)—including the International Hip Outcome Tool, the Copenhagen Hip and Groin Outcome Score, European Quality of Life-5 Dimensions Questionnaire, European Quality of Life Visual Analog Scale, the Hip Sports Activity Scale, visual analog scale for overall hip function, and a single question regarding satisfaction with treatment—were used as secondary outcomes.

**Results:**

Comparison of revision rates revealed a statistically significant decline from 9.1% for patients treated between 2012 and 2013 (n = 571 patients) to 1.2% for patients treated between 2017 and 2018 (n = 485 patients) (*P* < .0001). However, no statistically significant differences were observed in changes in PROMs between the 2 cohorts.

**Conclusion:**

There was a significant decline in revision rates over time. No changes were observed in PROMs over time.

Femoroacetabular impingement syndrome (FAIS) is a common and well-established cause of hip dysfunction, primarily in a young and active population. It is characterized by deep groin pain, reduced range of motion (ROM), and radiological signs of asphericity of the femoral head with cam morphology about the head-neck junction.^4,15^ Cam morphology entails an abnormal shape of the femoral head-neck junction, and pincer morphology refers to an abnormal morphology of the acetabulum.^5^ In FAIS, the biomechanics of the hip joint are disrupted and can lead to injury to the cartilage and the labrum.^5,30^

Arthroscopic surgery is an established and common method to treat FAIS. In recent years, several randomized controlled trials have shown the efficacy of arthroscopic treatment for FAIS compared with nonsurgical treatment.^6,9,20,23,28,32^ The principle behind the surgical intervention is to mitigate the hip pain and improve the impingement-free ROM by attempting to recreate normal hip anatomy. Hip arthroscopy has been shown to yield favorable results and positive patient-reported outcome measures (PROMs); however, revision in the form of reoperation due to any cause is not uncommon after primary surgery.^11,22^ Revision rates of 0.8% to 11.6% have been described in a limited number of studies, with no studies reporting on changes in revision rates over time.^2^ Little is known regarding whether these results have improved over time in this relatively young and rapidly developing field. With increasing use and experience with the arthroscopic method as a treatment for FAIS, there is a need for studies evaluating the changes in outcomes over time.

The primary aim of this study was to evaluate whether the outcomes after hip arthroscopy for FAIS have changed over time, using the revision rate as the primary outcome and PROMs as the secondary outcome. The primary hypothesis was that the revision rate would decline over time.

## Methods

Data were prospectively collected in a local hip arthroscopic registry and retrospectively reviewed. The registry includes patients treated with hip arthroscopy at 2 centers in Gothenburg, Sweden (Sahlgrenska University Hospital and GHP Ortho Center Gothenburg) since November 2011. Four surgeons (M.S.) were active during the 2012-2013 period. During the 2017-2018 period, the same 4 surgeons, along with 2 more surgeons (N.D.), were active, both of whom were trained by 1 of the other 4 surgeons.

The inclusion criteria for this study were patients who underwent hip arthroscopy for FAIS between January 2012 and December 2013 or January 2017 and December 2018 and were aged ≥18 years at the time of surgery, with no previous surgical intervention to either hip. Patient history, physical examination, and radiological findings were used to establish the diagnosis of FAIS.^8^ Intra-articular diagnostic injection was not routinely performed preoperatively if patients exhibited symptoms in accordance with FAIS supported by radiological findings. Indications for surgical treatment were patients diagnosed with FAIS who failed nonsurgical treatment before surgery. Patients receiving surgical interventions for indications other than FAIS were excluded. Patients who underwent surgery bilaterally were included and observed as 1 patient for PROMs but as 2 separate hips/operations for the revision rate and intraoperative data. The Tonnis grade was not reported in the registry.

Patients with bilateral surgical intervention and having received treatment at different dates for each hip were included in the study, provided their first hip surgery was registered in the hip arthroscopy register.

The revision rate was defined as an arthroscopic reoperation on the same hip within 2 years from the index surgery and recorded in the registry.

Patients were evaluated preoperatively and at the 2-year follow-up with the following PROMs: the Copenhagen Hip and Groin Outcome Score (HAGOS), European Quality of Life-5 Dimensions Questionnaire (EQ-5D), European Quality of Life Visual Analog Scale (EQ-VAS), the International Hip Outcome Tool (iHOT-12), the Hip Sports Activity Scale (HSAS), and a VAS for overall hip function.^10,24,29,34^ The scores have been validated and translated into Swedish.^14,27,33^ Furthermore, there was a single question regarding patient satisfaction at the 2-year follow-up. The minimally important change (MIC) and the Patient Acceptable Symptom State (PASS) were used to illustrate clinically relevant differences.^25^ The PASS cutoff values were based on findings in previous studies.^13,26^ In addition, each surgeon recorded intraoperative data.

The patients were divided into 2 groups. Group 1 underwent index surgery between 2012 and 2013, and group 2 underwent their index surgery between 2017 and 2018. The periods for the 2 groups were chosen based on the intention to include the first 2 full years of the registry and compare them with the last 2 years with available 2-year follow-up data.

This study was approved by the Swedish Ethical Review Authority (no. 2019-06050).

## Surgical Technique

All hip arthroscopies were performed in an outpatient setting and individualized according to the patient's pathology. The surgical technique used in this study has previously been presented by Sansone et al.^31^ With the patient in a supine position, an anterolateral portal and a mid-anterior portal were placed, and additional portals were created as needed. The peripheral compartment was visualized first by performing a longitudinal ligament-sparing capsulotomy of the anterior capsule. No transverse cut was used, and thus, no capsular closure was performed. Axial traction was applied to gain access to the central compartment of the hip joint. Pincer morphologies were resected using an “over-the-top” technique, leaving the labrum in situ when possible. In cases with larger pincer morphologies, the labrum was first detached and later reattached after rim resection. Labral debridement was performed if needed in those cases where degenerative changes were seen, but the labrum was still well attached to the acetabular rim. If the labrum attachment seemed compromised, then repair was performed regardless of the degenerative or traumatic cause. Resections of cam morphology were performed under the guidance of intraoperative fluoroscopy to help verify that the correct reshaping of the femoral head-neck junction had been achieved.

Microfracture was only considered in the setting of larger cartilage defects in the acetabulum, but most cases only showed smaller areas of chondral damage and were routinely debrided.

Patients were postoperatively permitted free ROM and weightbearing with the assistance of crutches for 4 weeks. Immediate postsurgery physical therapy was initiated, with the intensity increasing progressively. Emphasis was placed on improving strength, balance, endurance, coordination, and ROM. To minimize the risk of heterotopic ossification, the patients were prescribed nonsteroidal anti-inflammatory drugs the first 3 weeks after surgery.

## Statistical Analysis

Data were analyzed using IBM SPSS Statistics for Windows Version 28.0.1.1 (IBM Corp). Descriptive statistics were used for demographic characteristics and reported as the mean, standard deviation, median, and range, depending on the data distribution. The Pearson chi-square test was used to analyze the revision rate in group 1 compared with group 2. The Mann-Whitney *U* test was used to compare the intraoperative data and PROMs between the 2 groups. A nonparametric test was used for intraoperative data and PROMs because data were not normally distributed. The level of significance was set at *P* < .05.

The MIC was calculated using a distribution-based approach, corresponding to 0.5 SD of the change in the score. The PASS values have previously been established^13,26^ for the iHOT-12 as 67 and for the HAGOS subscores as follows: symptoms, 68.75; pain, 62.50; function in daily living, 82,50; sports, 60.94; physical activity, 43.75; and quality of life, 42.50.

A post hoc power analysis was calculated, which demonstrated 100% statistical power within the studied population regarding the difference in the revision rate.

## Results

Overall, 1056 patients in the local hip arthroscopy registry were eligible for the study. Group 1, treated with hip arthroscopy between January 2012 and December 2013, consisted of 571 (54%) patients with a mean age of 38 ± 13 years. Group 2, who had surgery between January 2017 and December 2018, consisted of 485 (46%) patients with a mean age of 34 ± 12 years. Accounting for bilateral treatment of the hips, the total number of treated hips was 1485 (100%), with 758 (51%) in group 1 and 727 (49%) in group 2. The loss to follow-up 2 years postoperatively regarding PROMs was 41%. Descriptive data are presented in Table 1.

**Table 1:** Patient Characteristics and Perioperative Data^*a*^

Characteristics	Group 1	Group 2	Difference	*P*
Total number of patients	571	485	86	
Total number of hips	758	727	31	
Operated side R/L/bilateral	47/33/20	42/31/27	5/2/7	
Male/female	68/32	76/24	8/8	**.003**
Day surgery	100	100	0	
Age, years	38 (13)	34 (12)	4	**<.0001**
Operation time, minutes	70.87 (17.97)	56.81 (21.38)	14.1	**<.0001**
Traction time, minutes	8.58 (7.58)	5.82 (3.38)	2.76	**<.0001**

a Data are presented as mean (SD) or %. Bold *P* values indicate statistical significance. L, left; R, right.

Out of all patients and hips treated in group 1, a total of 52 (9.1%) patients and 63 (8.3%) hips underwent a revision within 2 years of the primary surgery, compared with 6 (1.2%) patients and 7 (1%) hips in group 2. The change in the revision rate between the 2 groups, both regarding operated patients and hips, was significant (*P* < .0001). A significant reduction was observed in operation time and traction time between group 1 (70.87 ± 17.97 minutes and 8.58 ± 7.58 minutes) compared with group 2 (56.81 ± 21.38 minutes and 5.82 ± 3.38 minutes) (*P* < .0001). In addition, a significant reduction in the number of labral suture/resection (59/59 vs 5/15, *P* < .0001) and microfracture (35 vs 5, *P* < .0001) procedures were performed in the latter group. Intraoperative data and revision data are presented in Tables 1 to 3.

**Table 2:** Type of Arthroscopic Procedure Performed in All Hips in the 2 Groups^*a*^

Surgical Procedure	Group 1	Group 2	Difference	*P*
Femoral osteoplasty + acetabular osteoplasty (combined)	478	477	82	.305
Femoral osteoplasty	221	215	36	.860
Acetabular osteoplasty	4	6	2	.483
Labral suture	59	5	54	**<.0001**
Labral resection	59	15	44	**<.0001**
Microfracture	35	5	30	**<.0001**
Ligamentum teres resection	0	1	1	.307

a Data are presented as n. Bold *P* values indicate statistical significance.

**Table 3:** The Number and Percentage of Patients/Hips That Underwent Revision Surgery Out of All Patients/Hips in the 2 Groups^*a*^

Revision	Group 1	Group 2	Difference	*P*
Number of patients	52 (9.1)	6 (1.2)	46 (7.9)	**<.0001**
Number of hips	63 (8.3)	7 (1)	56 (7.3)	**<.0001**

a Data are presented as mean (SD). Bold *P* values indicate statistical significance.

Out of all patients, 672 (59%) answered all questionnaires before treatment and during the 2-year follow-up—360 patients in group 1 and 312 patients in group 2. Regarding PROMs, both groups reported higher outcomes postoperatively compared with preoperatively, but there was no significant difference in changes preoperatively compared with postoperatively between the groups. The percentage of patients achieving the MIC and PASS varied between 52% and 64% and 46% and 72% in group 1 compared with 56% and 72% and 46% and 73% in group 2; however, changes were not significant (Tables 4 and 5).

**Table 4:** Percentage of All Patients Exceeding the MIC Values for All Patients Answering All Questionnaires in the 2 Groups^*a*^

MIC Value	Group 1	Group 2	Difference	*P*
HAGOS				
Symptoms	58	70	12	.132
Pain	63	68	5	.097
Activities of daily living	58	63	5	.161
Sports	62	64	2	.282
Physical activity	56	61	5	.209
Quality of life	64	70	6	.098
iHOT-12	52	56	4	.847

a HAGOS, Copenhagen Hip and Groin Outcome Score; iHOT-12, International Hip Outcome Tool; MIC, Minimal important change.

**Table 5:** Percentage of All Patients Exceeding the PASS Values for All Patients Answering All Questionnaires in the 2 Groups^*a*^

PASS Value	Group 1	Group 2	Difference	*P*
HAGOS				
Symptoms	53	54	1	.666
Pain	72	73	1	.674
Activities of daily living	46	46	0	.934
Sports	56	59	3	.454
Physical activity	63	65	2	.483
Quality of life	66	66	0	.829
iHOT-12	51	52	1	.770

a HAGOS, Copenhagen Hip and Groin Outcome Score; iHOT-12, International Hip Outcome Tool; PASS, Patient Acceptable Symptom State.

Regarding satisfaction, 81% of patients in both groups reported being satisfied with the surgery, with no significant difference between the 2 groups (Tables 6 to 8).

**Table 6:** Preoperative PROMs for All Patients Answering All Questionnaires in the 2 Groups^*a*^

PROMs	Group 1	Group 2	Difference	*P*
HAGOS				
Symptom	51.0 (18.6)	49.1 (16.9)	1.9	.213
Pain	54.5 (19.3)	53.7 (18.5)	0.8	.740
Activities of daily living	46.6 (29.5)	47.4 (27.1)	0.8	.736
Sports	39.7 (22.2)	39.7 (21.9)	0	.998
Physical activity	29.4 (27.1)	25.6 (23.8)	3.8	.159
Quality of life	30.8 (17.2)	28.9 (15.3)	1.9	.209
EQ-5D	0.56 (0.30)	0.54 (0.30)	0.02	.113
EQ-VAS	65.3 (20.9)	62.8 (19.7)	2.5	**.011**
HSAS	3 (2.61)	2.76 (2.27)	0.24	.328
iHOT-12	42.2 (20.6)	43.1 (17)	0.9	.525
VAS: hip function	48.8 (21.2)	45.3 (20.5)	3.5	**.031**

a Data are presented as mean (SD). Bold *P* values indicate statistical significance. EQ-5D, European Quality of Life-5 Dimensions Questionnaire; EQ-VAS, European Quality of Life Visual Analog Scale; HAGOS, Copenhagen Hip and Groin Outcome Score; HSAS, Hip Sports Activity Scale; iHOT-12, International Hip Outcome Tool; VAS, visual analog scale.

**Table 7:** Postoperative PROMs for All Patients Answering All Questionnaires in the 2 Groups^*a*^

PROMs	Group 1	Group 2	Difference	*P*
HAGOS				
Symptoms	68.5 (21.4)	67.7 (21.4)	0.8	.649
Pain	74.4 (21.8)	74.4 (20.8)	0	.731
Activities of daily living	71.7 (28.4)	72.7 (27.6)	1	.778
Sports	64.2 (28.9)	64 (26.8)	0.2	.634
Physical activity	55.8 (33.8)	56 (33)	0.2	.976
Quality of life	57.2 (28.2)	57.1 (28)	0.1	.961
EQ-5D	0.73 (0.30)	0.68 (0.35)	0.05	.066
EQ-VAS	74.4 (19.1)	71.7 (18.1)	2.7	**.006**
HSAS	3.40 (2.06)	3.55 (2.16)	0.15	.377
iHOT-12	59.8 (31.2)	59.6 (29.3)	0.2	.601
VAS: hip function	69.2 (22.9)	66.1 (22.6)	3.1	**.030**
Satisfaction	81	81		

a Data are presented as mean (SD) or %. Bold *P* values indicate statistical significance. EQ-5D, European Quality of Life-5 Dimensions Questionnaire; EQ-VAS, European Quality of Life Visual Analog Scale; HAGOS, Copenhagen Hip and Groin Outcome Score; HSAS, Hip Sports Activity Scale; iHOT-12, International Hip Outcome Tool; PROMs, patient-reported outcomes measures; VAS, Visual Analog Scale.

**Table 8:** Changes in PROMs From Pre- to Postoperative for All Patients Answering All Questionnaires in the 2 Groups^*a*^

PROMs	Group 1	Group 2	Difference	*P*
HAGOS				
Symptoms	17.5 (22.4)	18.6 (20.7)	1.1	.301
Pain	19.9 (23.5)	20.6 (21.2)	0.7	.586
Activities of daily living	25.1 (31.6)	25.2 (27.9)	0.1	.636
Sports	24.4 (30.6)	24.3 (27.2)	0.1	.810
Physical activity	26.4 (36.2)	30.4 (35.8)	4	.093
Quality of life	26.4 (28.2)	28.2 (26.2)	1.8	.315
EQ-5D	0.17 (0.36)	0.14 (0.41)	0.03	.670
EQ-VAS	9.21 (22)	9.17 (21.5)	0.04	.949
HSAS	0.42 (2.60)	0.79 (2.27)	0.37	.060
iHOT-12	17.6 (31.8)	16.5 (31.2)	0.9	.924
VAS: hip function	20.7 (27.2)	20.8 (25.3)	0.1	.981

a Data are presented as mean (SD). Bold *P* values indicate statistical significance. EQ-5D, European Quality of Life-5 Dimensions Questionnaire; EQ-VAS, European Quality of Life Visual Analog Scale; HAGOS, Copenhagen Hip and Groin Outcome Score; HSAS, Hip Sports Activity Scale; iHOT-12, International Hip Outcome Tool; PROMs, patient-reported outcomes measures; Pre, preoperative; VAS, Visual Analog Scale.

## Discussion

The main finding in this retrospective observational registry study with prospectively collected data was a marked decrease over time in the revision rate in patients treated for FAIS, with simultaneous significant improvements in intraoperative markers, such as shorter operation time and traction time.

No studies have investigated the changes in the revision rate over time. However, West et al^35^ reported revision rates for 2012 and 2013, with a revision rate of 4% in patients who underwent hip arthroscopy for FAIS between 2007 and 2015. Disegni et al^3^ showed a revision rate of 8.2% between 2008 and 2014 during a 5-year follow-up. No studies reporting revision rates between 2017 and 2018 were found.

Compared with the present study, these studies had longer follow-ups. However, West et al^35^ reported that 86% of all revisions occurred within the first 18 months postoperatively, thus making the comparison with this 2-year follow-up more applicable. One systematic review by Go et al^7^ reported a correlation between a surgeon's experience, both operatively and in the selection of patients, and the revision rate, which could be one explanation for the lower revision rate in group 2 compared with group 1 in our study. The significantly lower revision rate in group 2, together with a shortened surgical time, suggests that the revision rate could serve as an objective measurement of improvement in the care of patients with FAIS.

The number of surgeries performed decreased in group 2, likely due to increased understanding of the pathology of FAIS, as well as increased experience in the selection of patients benefitting from arthroscopic intervention. Together with increased experience in performing the arthroscopic surgery among the surgeons, this could, in turn, result in a decreased revision rate.

Secondary outcomes in the present study consisted of comparing the change in PROMs pre- and postoperatively, along with the number of patients exceeding MIC and PASS values. The present study demonstrated improvements in PROMs within the groups, which is in accordance with results from previous studies.^9,18,19,21^ However, comparisons between the groups demonstrated no significant difference.

The surgical technique evolved somewhat between the aforementioned periods. Hip-distraction time significantly reduced as our techniques evolved, allowing us adequate access to the acetabular rim and femoral neck and only distracting the hip to observe and, if needed, treat labral or cartilage pathology. In addition, minor adjustments to portal positions and more effective use of intraoperative fluoroscopy are some of the additional changes over the years. The number of labral procedures decreased significantly in this study. This reflects a growing belief at our institution that the primary pathology in patients with cam-type FAI most often is a resultant cartilage lesion and is treated as such.^1,12^

Strengths of the present study include prospective data collection, a large and consecutive cohort, and data from the early stages of the implementation of arthroscopic treatment for FAIS in this particular geographic region. Furthermore, relatively few surgeons performed all the procedures during the 2 time periods; 4 out of the total of 6 surgeons conducted hip arthroscopies during both periods, making the results less biased toward individual differences besides improved experience.

The registry's limitations include no data regarding body mass index and the Tonnis grade, and incomplete data regarding the Konan classification.

Limitations of the present study include a relatively large loss to follow-up in PROMs, with a 2-year follow-up rate of 59% in total. However, it has previously been demonstrated in this register that there was no significant difference between the nonresponders and the responders regarding PROMs during the 2-year follow-up.^17^ Loss of patients to conversion to total hip arthroplasty (THA) within the 2-year follow-up is not included, as these data were not available. However, 1 study previously conducted by Lindman et al^16^ compared the same local registry and matched it with a nationwide arthroplasty registry between the years 2011 and 2018 and found that out of 2516 patients, 135 patients (5.4%), with a mean age of 51 years, were converted to THA after hip arthroscopy. The mean time interval after arthroscopic hip surgery to conversion to THA was 27 months. The number of patients converted to THA, and therefore not included in the final analysis, would make up a considerable portion of the cohort and could affect the results. Patients in group 2 with persistent symptoms after hip arthroscopy, similar to patients in group 1 who underwent revision arthroscopy with poor outcomes, were likely recommended THA in favor of a revision arthroscopy by the same surgeons who were more experienced between 2017 and 2018, compared with 2012 and 2013. This could partly explain the difference in revision rates. Patients treated at other clinics are not included in the local hip arthroscopy registry, as a result, patients who potentially had arthroscopic revision surgery at another clinic were not accounted for. However, the patients are thoroughly observed, and due to the administrative circumstances in Sweden and the scarcity of hip arthroscopy surgeons, the risk that any revision arthroscopies are missed is deemed low.

## Conclusion

The revision rate, operation time, and traction time showed a significant decline over time. Simultaneously, no changes were observed in PROMs.
